# Baicalin Exerts Anti-Airway Inflammation and Anti-Remodelling Effects in Severe Stage Rat Model of Chronic Obstructive Pulmonary Disease

**DOI:** 10.1155/2018/7591348

**Published:** 2018-10-08

**Authors:** Genfa Wang, Nabijan Mohammadtursun, Yubao Lv, Hongying Zhang, Jing Sun, Jingcheng Dong

**Affiliations:** ^1^Department of Integrative Medicine, Huashan Hospital, Fudan University, Shanghai, China; ^2^The Institutes of Integrative Medicine, Fudan University, Shanghai, China; ^3^Department of TCM, The Second Affiliated Hospital of Nanchang University, Nanchang, China; ^4^College of Xinjiang Uyghur Medicine, Hotan, China

## Abstract

Chronic obstructive pulmonary disease (COPD) is a worldwide epidemic. Current approaches are disappointing due to limited improvement of the disease development. The present study established 36-week side stream cigarette smoke induced rat model of COPD with advanced stage feature and evaluted the effects of baicalin on the model. Fifty-four Sprague–Dawley rats were randomly divided into six groups including room air control, cigarette smoke exposure, baicalin (40 mg/kg, 80 mg/kg, and 160 mg/kg), and budesonide used as a positive control. Rats were exposed to cigarette smoke from 3R4F research cigarettes. Pulmonary function was evaluated and pathological changes were also observed. Cytokine level related to airway inflammation and remodelling in blood serum, bronchoalveolar lavage fluid, and lung tissue was determined. Blood gases and HPA axis function were also examined, and antioxidant levels were quantified. Results showed that, after treatment with baicalin, lung function was improved and histopathological changes were ameliorated. Baicalin also regulated proinflammatory and anti-inflammatory balance and also airway remodelling and anti-airway remodelling factors in blood serum, bronchoalveolar lavage fluid, and lung tissue. Antioxidant capacity was also increased after treatment with baicalin in COPD rat model. HPA axis function was improved in baicalin treated groups as compared to model group. Therefore, baicalin exerts lung function protection, proinflammatory and anti-inflammatory cytokine regulation, anti-airway remodelling, and antioxidant role in long term CS induced COPD model.

## 1. Introduction

Chronic obstructive pulmonary disease (COPD) is progressive and life-threatening lung disease which encompasses chronic obstructive bronchitis and emphysema and characterized by not fully reversible air flow limitations [1, 2]. COPD accounts for the majority of morbidity and mortality in the world. Smoking is major trigger factor for COPD [3]. Patients diagnosed with COPD are expected to grow rapidly in developing countries where tobacco use is the most prevalent and calls for new therapeutic approaches for the disease treatment.

Cigarette smoke (CS) is a mixture of more than 5000 chemical compounds which induces local inflammation and oxidative stress in the lung [4, 5]; inflammation is perpetuated by immune cells like macrophages and neutrophils as part of the inflammatory responses, particularly in severe COPD patients; once the process is activated, the disease will progress because of persistent inflammation which persists even after smoking cessation [6, 7]. Inflammation also increases by oxidative stress from inhaled smoke and arising protease-anti protease imbalance includes activation of metalloproteinase and inactivation of anti-protease [8, 9]. Thus, a cascade of inflammatory factors increases including proinflammatory cytokines such as IL-1*β*, IL-6, TNF-*α*, MMP-9, and IL-8 [10, 11]. In addition to inflammatory responses, airway remodelling is one of the main characteristics of COPD [12]. Whether airway remodelling is the consequence of airway inflammation is still arguable, but the long term exposure to proinflammatory mediators may accelerate the formation of chronic inflammation with damage and metaplasia of the airway epithelium [13], and airway remodelling factors were also increased. Therefore, restoring the balance between inflammatory and anti-inflammatory factors is one of the effective strategies to treat COPD.

Although COPD is believed to be common preventable and treatable disease, it still represents a common unmet therapeutic need worldwide. Most available treatments only abate COPD's debilitating flare-ups, and till now inhaled corticosteroids (ICS) and long-acting *β*-agonists (LABA) are frequently used to treat COPD, but they were proved to be not ideal due to corticosteroid resistance [3]. Regarding the increased incidence of the disease, exploring new effective treatments for COPD would be of a great significance.

Previous studies have reported that flavonoid baicalin, extracted from dried roots of* Scutellaria baicalensis*, has anti-inflammatory effects in short term cigarette smoke induced airway inflammation [14, 15] and anti-airway remodelling in asthmatic mice model [16]. It was also reported that baicalin also exerts anticancer effects in different types of cancers [17, 18], indicating the potential role for the treatment of airway inflammation disease, such as COPD. However, the potential of baicalin for anti-inflammatory benefits in addition to chronic inflammation in severe stage or long term smokers is not well elucidated. In this study, we used baicalin and evaluated its anti-inflammatory, antioxidant, and anti-airway remodelling effects in 36 wk CS induced severe COPD rat model

## 2. Material and Methods

### 2.1. Reagents

Baicalin was purchased from Chengdu Mansite Ansite Bio-technology (Sichuan, China). Budesonide was purchased from Astrazeneca. 3R4F research cigarettes were purchased from the University of Kentucky (Lexington, KY, USA); pentobarbital sodium was purchased from Merck (Darmstadt, Germany), and enzyme-linked immunosorbent assay (ELISA) kits used in this study were purchased from R&D Systems (Minneapolis, MN, USA).

### 2.2. Animals

Fifty-four Male Sprague-Dawley (SD) rats, two months old, weighing 200 ± 20 g, were provided by Xipuer–Bikai Laboratory Animal Co., Ltd. (Shanghai, China; license number, SCXK [Hu]2008-0016). After one week of accommodation, rats were randomly divided into 6 groups (8-9 rats/group) and placed into laboratory in the animal center of School of Pharmacy, Fudan University, at 22 ± 2°C with a 12-h light/dark cycle and 55 ± 5% humidity. Control group was exposed to room air (RA), and model group (M) and low (LB), middle (MB), high dosage of baicalin (HB) group, and positive control (PC) were exposed to second-hand smoke with a period of 36 weeks. All procedure were carried out in compliance with the institutional review board of Shanghai Medical College of Fudan University (permit number: SYXK (hu)2010–0099) and in accordance with NIH guide for animal use of laboratory animals, China.

### 2.3. COPD Model Establishment

COPD model establishment was performed as previously described [19]. Briefly, all rats were exposed to side-stream cigarette smoke from 3R4F reference cigarettes with total particular matter 10.9 mg/cigt, tar 9.4 mg/cigt, nicotine 0.726 mg/cigt, and carbon monoxide 12 mg/cigt: seven cigarettes for one hour/3 times a day, 6 days a week. Buxco smoking apparatus was used with the smoking chamber (Buxco, NC, USA). The exposure period was 36 weeks. The rats in control group and COPD model group were treated with normal saline water, and rats in LB, MB, and HB group were treated by intragastric administration of 40 mg/kg/d, 80 mg/kg/d, and 160 mg/kg/d baicalin, respectively. Rats in positive control group were treated with budesonide (0.2 mg/kg/d) by intragastric administration. Both CS exposure and drug administration followed for 36 weeks. And on day 253, lung function evaluation was conducted before rats were sacrificed for sample collection

### 2.4. Lung Function Evaluation

2% pentobarbital (40 mg/kg) was intraperitoneally injected for anesthetization of the rats and all rats were tracheostomised and placed in a forced pulmonary manoeuvre system which was connected to a computer (Buxco, NY, USA) and imposed to an average breathing frequency of 150 breaths/min. Rats were forced to breathe against a closed valve at the mouth through which functional residual capacity (FRC) could be determined. The lungs were inflated to a standard pressure of +30 cm H_2_O total lung capacity (TLC) and then slowly deflated until a pressure of −30 cm H_2_O was achieved. Different lung volumes, such as the forced expiratory volume in 1 s (FEV1) and forced vital capacity (FVC), were then recorded by computer software.

### 2.5. Blood Gas Analysis

Blood gas analysis was conducted according to the GEM3000 blood gas analyzer operation instructions. Briefly, rats were anesthetized and blood samples were taken, inserting 25 IU/ml heparin sodium to the blood sample, before determination samples should be well mixed.

### 2.6. Tissue Preparations

The lungs were harvested and stored at -80°C for histological observation and following analysis. Blood samples and bronchoalveolar lavage fluid (BALF) were collected as previously described [14, 19]. Briefly Blood samples were stored at 4°C prior to centrifugation at 5000 ×g for 15 min and preserved at -80. For BALF collection, the trachea was cannulated and the bronchoalveolar was lavaged with 3 ml phosphate buffered solution (PBS) three times. BALF samples were centrifuged at 500 g for 5 min at 4°C and the supernatant was stored at −80°C.

### 2.7. Histopathological Examination

Lungs were extracted and fixed in 10% formalin for 24 h. Paraffin-embedded tissues were cut into 4 mm thick sections, placed on poly-L-lysine-coated slides, and then incubated for 24 hours at 58°C. Deparaffinized sections were stained with hematoxylin and eosin (H&E). Light microscope was used to visualize lung tissue pathological changes.

### 2.8. Measurements of Inflammatory Factors

The levels of IL-6, IL-8, IL-10, IL-1*β*, IL-17, TNF-*α*, TFG-*β*1, MMP-2, TIMP-1, and MMP-9 were measured in serum. IL-6, IL-8, IL-10, TNF-*α*, IL-1*β*, and IL-17 content in BALF were determined. Lung tissue IL-6, IL-8, IL-10, and TNF-*α* levels were also measured, by using commercially available ELISA assay kit according to the manufacturer's instruction.

### 2.9. Measurement of MDA, SOD, and HO-1 in Serum

The activities of MDA, SOD, and HO-1 in serum were measured as described in the manufacturer's protocol (R&D Systems, Minneapolis, MN, USA). Measurement of ATCH, CRH, corticosterone, leptin in blood serum: the level of ATCH, CRH, corticosteroid, and leptin were determined in serum using ELISA assay kits (R&D Systems, Minneapolis, MN, USA) according to the manufacturer's instruction.

### 2.10. Statistical Analysis

Experimental data were expressed as means ± standards deviation (SDs). Differences between groups were determined by one-way ANOVA with the Tukey multiple comparison test. P values < 0.05 were considered to be significant. Calculations were made using Graphpad Prism.

## 3. Results

### 3.1. Baicalin Improved Lung Function in Rat Model of COPD

**Figure 1:**
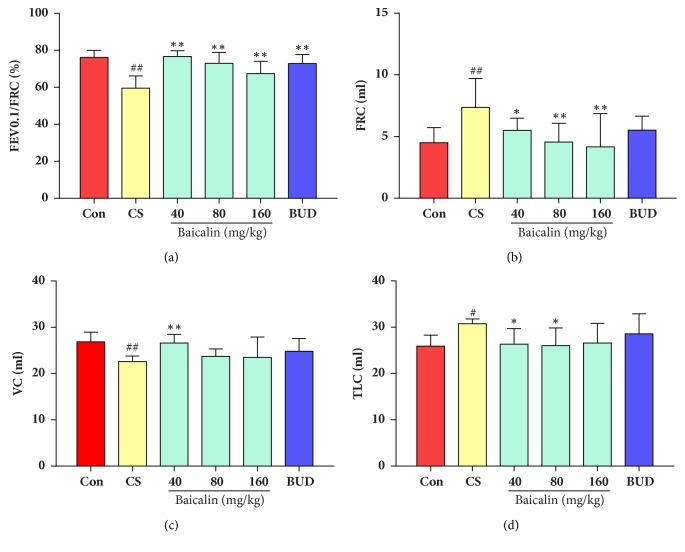
Effects of baicalin on pulmonary function of COPD rat model. (a) The FEV0.1/FVC ratio. (b) FRC. (c) Vital capacity (VC). (d) The TLC level. The data are shown as the mean ± the standard deviation (SD). ^*∗*^p < 0.05 and ^*∗∗*^p < 0.01 indicate a statistically significant difference compared to the model group; ^#^p < 0.05 and ^##^p < 0.01 indicate a statistically significant difference in model group compared with control group. FEV0.1, forced expiratory volume in 0.1 s; FRC, functional residual capacity; FVC, forced vital capacity; MMEF, maximum mild expiratory flow; TLC, total lung capacity.

Measurements of lung function indicators in 36-week CS exposure rat model are shown in Figure 1. The FEV_0.1_/FRC ratio and vital capacity (VC) were significantly decreased while FRC and TLC were markedly higher in 36-week CS exposure model group compared to control group (p < 0.05, p < 0.01). CS induced rats treated with different concentrations of baicalin and positive control displayed an increased FEV_0.1_/FRC ratio compared with model group (P < 0.01, Figure 1(a)), while 40 mg/kg baicalin raised VC value of CS exposure rats compared with model group (p < 0.01, Figure 1(c)), and there is no difference between 80 mg/kg, 160 mg/kg baicalin treated group and positive control compared to model group (p > 0.05, Figure 1(c)). Functional residual capacity (FRC) was significantly decreased in all groups treated with baicalin compared to model group (p < 0.01, p < 0.05.  Figure 1(b)) and total lung capacity (TLC) was significantly decreased in low and middle dosage of baicalin groups compared to model group (p < 0.05, Figure 1(d)). However, FRC and TLC value in positive control had no difference to model group (p > 0.05, Figures 1(b) and 1(d)). These results indicated that baicalin exerts a lung function protection role in cigarette smoke COPD rats.

### 3.2. Histopathology Changes

**Figure 2:**
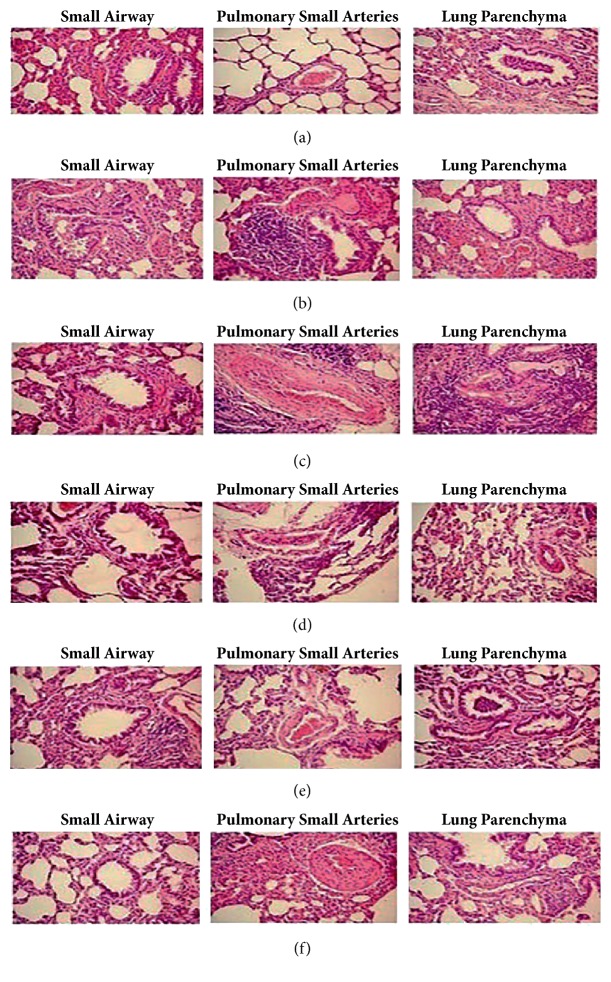
Photograph of HE-stained lung tissue under an optical microscope (×200). (a) control group; (b) COPD model group; (c) 40 mg/kg baicalin treated group; (d) 80 mg/kg baicalin treated group; (e) 160 mg/kg baicalin treated group; (f) budesonide treated group. CS=cigarette smoking; BUD=budesonide.

**Figure 3:**
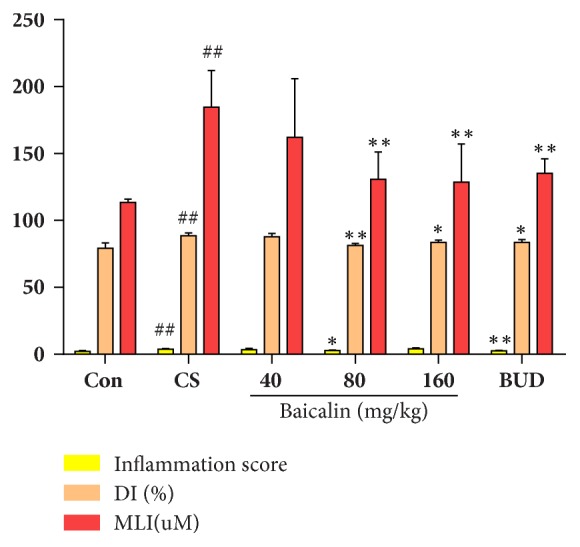
Inflammation score, destructive index, and mean linear intercepts. The data are shown as the mean ± the standard deviation (SD). ^*∗*^p < 0.05 and ^*∗∗*^p < 0.01 indicate a statistically significant difference compared to the model group; ^#^p < 0.05 and ^##^p < 0.01 indicate a statistically significant difference in model group compared with control group. H&E, haematoxylin–eosin; DI, destructive index; MLI, mean linear intercepts.

We stained pulmonary tissue with H&E before pathological examination. Results showed that inflammatory cells infiltration was more evident in tissues of small airways, pulmonary small arteries, and lung parenchyma in cigarette smoke exposure rats compared with control groups (Figures 2(a) and 2(b)) and corresponding phenomena was relieved in baicalin treated groups in different level compared with model group (Figures 2(c), 2(d), and 2(e)). Moreover, inflammation related parameters were also analyzed according to HE staining results; our data showed that inflammatory scores, mean linear intercepts (MLI), and destructive indexes (DI) were significantly higher in model group compared to control group (p < 0.05, p < 0.01 respectively, Figure 3). In CS exposure groups treated with middle (80 mg/kg) and high dosage (160 mg/kg) of baicalin, budesonide significantly reduced DI and MLI degree as compared to model group (p < 0.05, p < 0.01, respectively, Figure 3), while lower inflammation score was measured.

### 3.3. Effects of Baicalin on Cytokine Levels in Blood Serum

**Figure 4:**
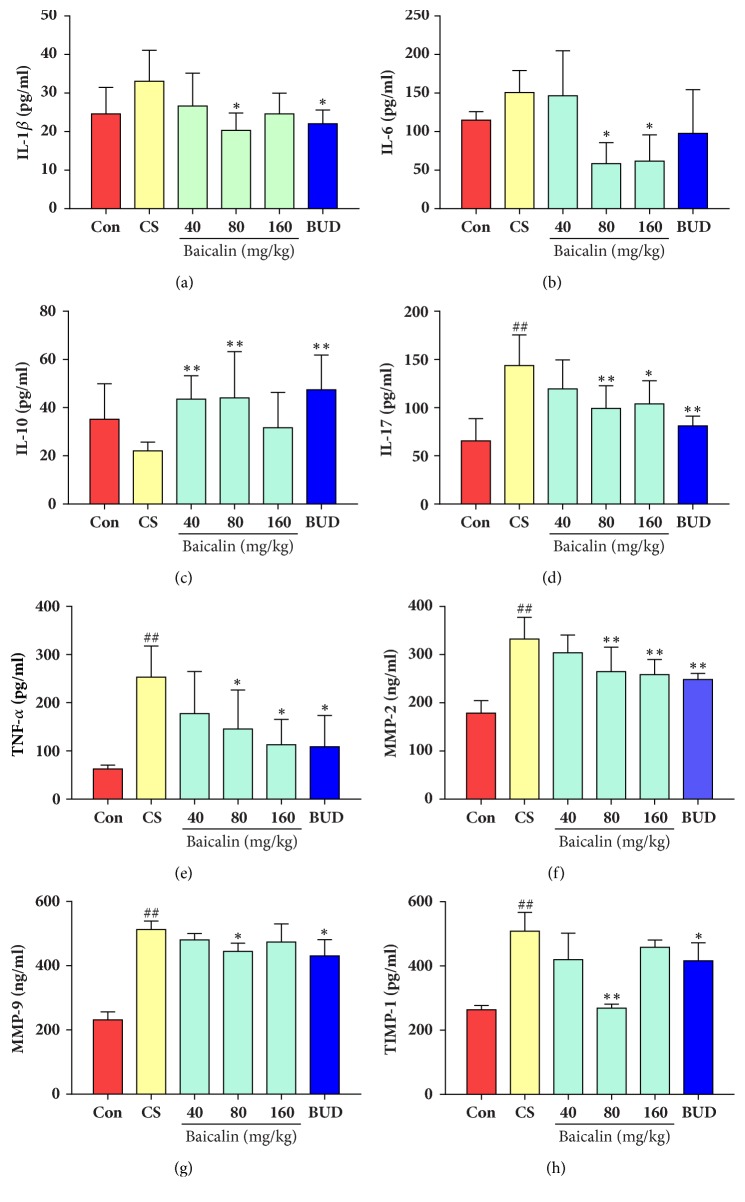
Effects of baicalin on cytokine changes in blood serum. (a) IL-1*β*; (b) IL-6; (c) IL-10; (d) IL-17; (e) TNF-*α*; (f) MMP-2; (g) MMP-9; (h) TIMP-1. The data are shown as the mean ± the standard deviation (SD). ^*∗*^p < 0.05 and ^*∗∗*^p < 0.01 indicate a statistically significant difference compared to the model group; ^#^p < 0.05 and ^##^p < 0.01 indicate a statistically significant difference in model group compared with control group. IL, interleukin; TNF, tumour necrosis factor; MMP, metalloproteinase; TIMP, tissue inhibitor of metalloproteinase.

Rat cytokine levels were quantified in blood serum; as shown in Figure 4, there was no significant change observed in the level of IL-1*β*, IL-6, and IL-10 between control group and model group (p > 0.05, Figures 4(a), 4(b), and 4(c)), while model group displayed higher TNF-*α*, IL-17, MMP-2, MMP-9, and TIMP-1 levels compared with control group (p < 0.05, p < 0.01, respectively, Figures 4(d), 4(e), 4(f), 4(g), and 4(h)). In CS exposure groups treated with middle (80 mg/l) and high (160 mg/kg) dosage of baicalin and budesonide IL-6, IL-17, TNF-*α*, and MMP-2 levels in blood serum markedly reduced compared to model groups (p < 0.05, p < 0.01, respectively, Figures 4(b), 4(d), 4(e), and 4(f)). There was also significant difference in the level of IL-1*β*, MMP-9, and IL-10 in middle dosage of baicalin (80 mg/kg) and budesonide treated group compared to controls (p < 0.05, p < 0.01, respectively, Figures 4(a), 4(c), and 4(g)). Vascular endothelial growth factor and TGF-*β* concentration were also recorded. Results showed that VEGF level was increased after CS treatment (p < 0.05, Supplemental Figure 1(A)), and low level of baicalin intervention significantly reduced VEGF concentration (p < 0.05), while increased TGF-*β* content was not changed after baicalin intervention (p > 0.05, p < 0.05, Supplemental Figure 1(B)). However, budesonide intervention decreased both VEGF and TGF-*β* level (p < 0.05, p < 0.05, Supplemental Figures 1(A) and 1(B))

### 3.4. Effect of Baicalin on Antioxidant Activity and Lipid Peroxidation

**Figure 5:**
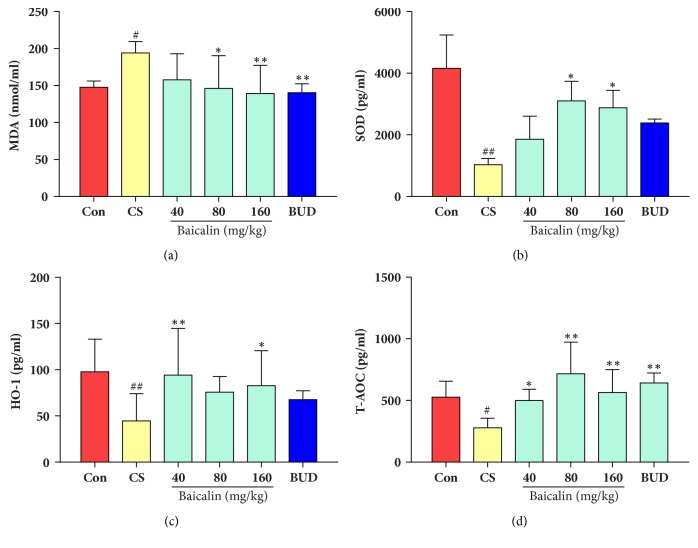
Effects of baicalin on lipid peroxidation and antioxidants in blood serum. (a) MDA; (b) SOD; (c) HO-1; (d) T-AOC. The data are shown as the mean ± the standard deviation (SD). ^*∗*^p < 0.05 and ^*∗∗*^p < 0.01 indicate a statistically significant difference compared to the model group; ^#^p < 0.05 and ^##^p < 0.01 indicate a statistically significant difference in model group compared with control group. MDA, malondialdehyde; SOD, superoxide dismutase; HO-1, heme oxygenase; T-AOC, total antioxidant capacity.

In COPD model groups, the content of MDA in blood serum was significantly higher compared with that in control group (p < 0.05), while significant reduction in MDA content was measured in groups treated with baicalin (80, 160 mg/kg) compared with model group (p < 0.05, p < 0.01 respectively, Figure 5(a)); positive control budesonide also decreased the level of MDA compared with that in model group (p < 0.01), indicating that baicalin significantly reduced MDA production, outcome of lipid peroxidation.

Total antioxidant capacity, superoxide dismutase, and heme-oxygenase level were also measured. Results showed that T-AOC, SOD, and HO-1 significantly reduced in 36-week CS exposure COPD model group blood serum as compared to controls (p < 0.05, p < 0.01 respectively, Figures 5(b), 5(c), and 5(d)). By contrast, total antioxidant capacity was increased in CS exposure groups treated with baicalin and budesonide treated groups compared with that in control groups (p < 0.05, p < 0.01, respectively, Figure 5(d)). In addition, SOD content in blood serum of rats treated with baicalin (80, 160 mg/kg) significantly increased compared to model groups (p < 0.05, p < 0.01, respectively, Figure 5(b)), Furthermore, the treatment with baicalin (40, 160 mg/kg) significantly enhanced HO-1 production compared with those in model group (p < 0.05, p < 0.01, respectively, Figure 5(b)). These results suggested that baicalin could improve antioxidant capacity and prevent lipid peroxidation in CS induced model of COPD.

### 3.5. Effects of Baicalin on Blood Gas

**Figure 6:**
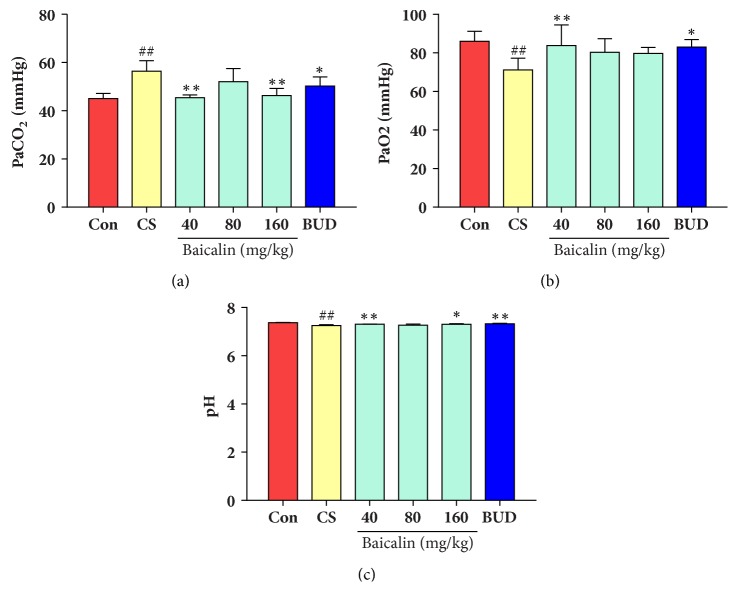
Effect of baicalin on arterial blood gas change in CS induced COPD rat model. (a) PaCO2; (b) PaO2; (c) pH. The data are shown as the mean ± the standard deviation (SD). ^*∗*^p < 0.05 and ^*∗∗*^p < 0.01 indicate a statistically significant difference compared to the model group; ^#^p < 0.05 and ^##^p < 0.01 indicate a statistically significant difference in model group compared with control group.

To evaluate the effects of baicalin on CS exposed rat model of COPD, blood gas was also analyzed. After 36-week CS exposure, blood pH and PaO_2_ were significantly decreased while PaCO_2_ content (Figure 6(c)) markedly increased as compared to control group (p < 0.01, Figures 6(a), 6(b), and 6(c)). However, administration of 40 mg/kg, 160 mg/kg baicalin markedly prevented PaCO_2_ augmentation and increased pH level compared to model group (p < 0.05, p < 0.01, Figures 6(a), and 6(c)). There was also significant increase in PaO_2_ level after treatment with 40 mg/kg baicalin compared to model group (P < 0.05). These results demonstrated that baicalin can effectively control physiologic parameters of CS-induced blood gas change in COPD rat model.

### 3.6. Effects of Baicalin on Inflammatory Cytokine Levels in Bronchoalveolar Lavage Fluid and Lung Tissue

**Figure 7:**
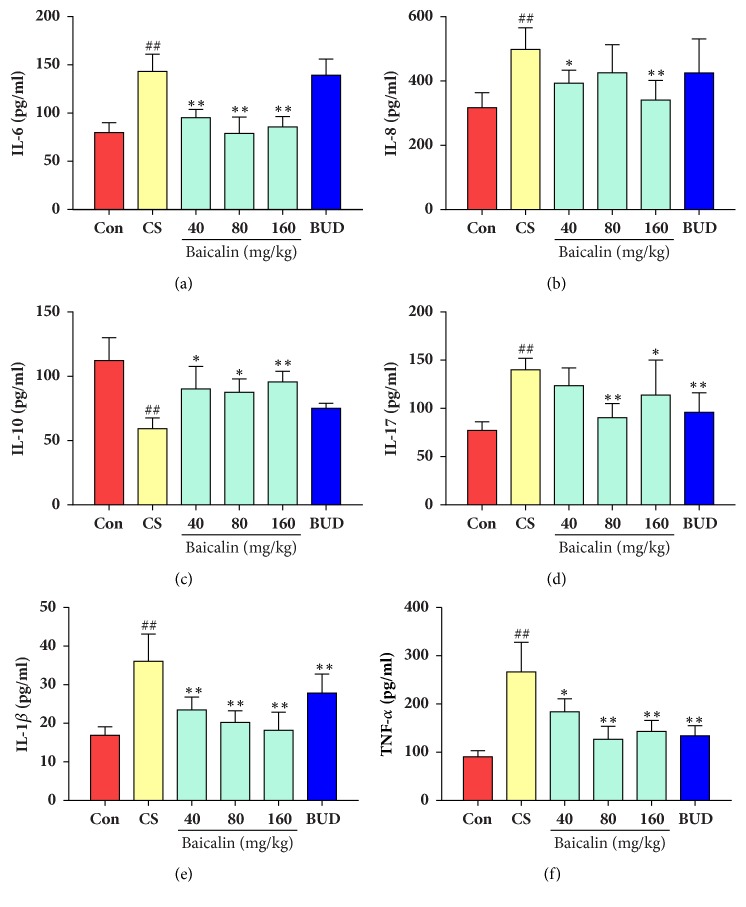
Effects of baicalin on inflammatory cytokine change in bronchoalveolar lavage fluid. (a) IL-6; (b) IL-8; (c) IL-10; (d) IL-17; (e) IL-1*β*; (f) TNF-*α*. The data are shown as the mean ± the standard deviation (SD). ^*∗*^p < 0.05 and ^*∗∗*^p < 0.01 indicate a statistically significant difference compared to the model group; ^#^p < 0.05 and ^##^p < 0.01 indicate a statistically significant difference in model group compared with control group. IL-6, interleukin-6; IL-8, interleukin8; IL-10, interleukin-10; IL-17, interleukin-17; IL-1*β*, interleukin-1*β*; TNF-*α*, tumour necrosis factor-*α*.

As shown in Figure 7, inflammatory cytokines in bronchoalveolar lavage fluid cytokine levels were also determined. Results showed that IL-6, IL-8, IL-17, IL-1*β*, and TNF-*α*, level were significantly higher while IL-10 content declined in 36-week CS induced COPD rat model group as compared to those in control (p < 0.01). However, baicalin (40, 80, 160 mg/kg) effectively prevented IL-6 (Figure 7(a)), IL-1*β* (Figure 7(e)), and TNF-*α* (Figure 7(f)) level amplification and also upregulated IL-10 level as compared to model group (p < 0.05, p < 0.01, respectively). IL-8 level was also declined in baicalin (40, 160 mg/kg) treated groups (Figure 7(b)) while it was effective in preventing IL-17 increase in 80 mg/kg and 160 mg/kg baicalin (Figure 7(d)) treated groups as compared to control (p < 0.05, p < 0.01).

**Figure 8:**
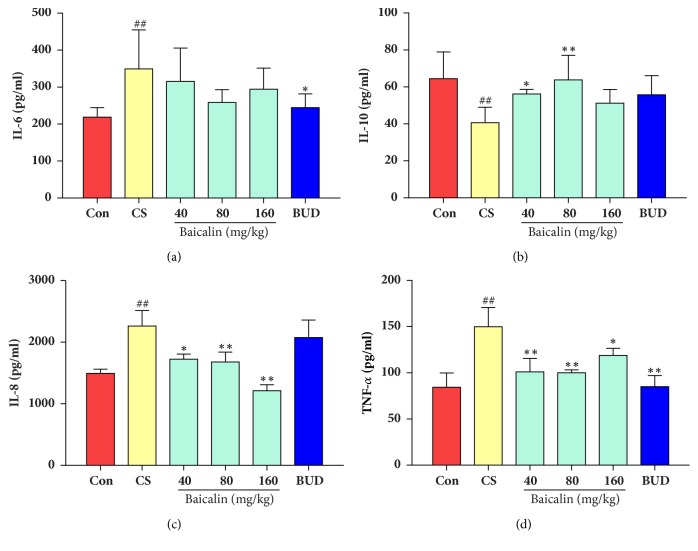
Effects of baicalin on inflammatory cytokine change in lung tissue. (a) IL-6; (b) IL-10; (c) IL-8; (d) TNF-*α*. The data are shown as the mean ± the standard deviation (SD). ^*∗*^p < 0.05 and ^*∗∗*^p < 0.01 indicate a statistically significant difference compared to the model group; ^#^p < 0.05 and ^##^p < 0.01 indicate a statistically significant difference in model group compared with control group. IL-6, interleukin-6; IL-8, interleukin8; IL-10, interleukin-10; TNF-*α*, tumour necrosis factor-*α*.

Inflammatory cytokines in lung tissue were also tested; results showed that IL-6, IL-8, and TNF-*α* content in lung tissue of COPD rat model significantly increased compared to control (p < 0.01, Figures 8(a), 8(c), and 8(d)). There was a descending trend, but no significant change was observed in IL-6 level (p>0.05) after treating with baicalin (40, 80, and 160 mg/kg) while significant decline was found in IL-8 and TNF-*α* content compared to those in model group (p < 0.05, p < 0.01, Figures 8(a), 8(c), and 8(d)). By contrast, IL-10 level was markedly increased in CS exposure groups treated with baicalin (40 and 80 mg/kg) as compared to model groups (p < 0.05, p < 0.01, Figure 8(b)). It was found that, in budesonide group, there was no significant difference in IL-10 and IL-8 level (p > 0.05, (Figures 8(b) and 8(c)), while TNF-*α* and IL-6 significantly decreased as compared to model group (p < 0.01, Figures 8(a) and 8(d)). The above results indicated that baicalin reduced proinflammatory cytokines level in lung tissue.

### 3.7. Effects of Baicalin on HPA Axis Function

**Figure 9:**
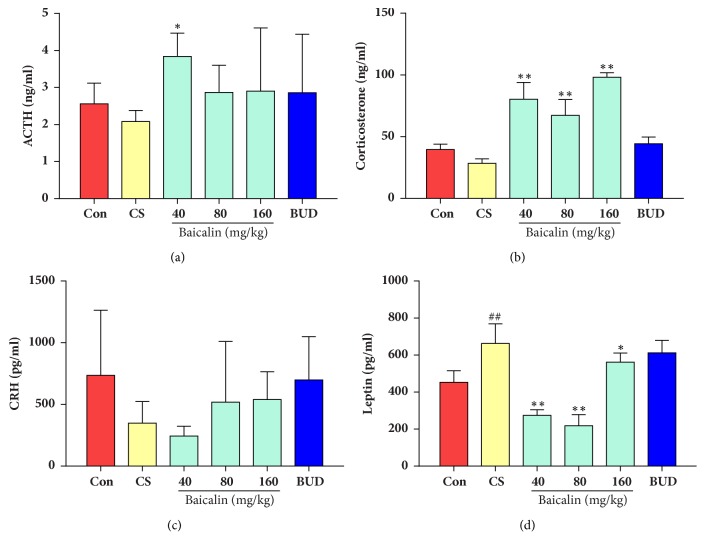
Effects of baicalin on HPA axis function related factors. (a) ACTH; (b) corticosteroid; (c) CRH; (d) leptin. The data are shown as the mean ± the standard deviation (SD). ^*∗*^p < 0.05 and ^*∗∗*^p < 0.01 indicate a statistically significant difference compared to the model group; ^#^p < 0.05 and ^##^p < 0.01 indicate a statistically significant difference in model group compared with control group. HPA, hypothalamic-pituitary-adrenal axis; ACTH, adreno-cortico-tropic-hormone; CRH, corticotropin releasing hormone.

To examine the effects of HPA axis function, corticosteroid, adrenocorticotropic hormone (ACTH), corticotropin releasing hormone (CRH), and leptin content were measured. Results showed that there were descending trends in ACTH, corticosteroid, and CRH level in model group without statistical significance (p > 0.05), while leptin level significantly increased compared to control (p < 0.05). However, after treatment with baicalin, corticosterone level effectively increased while leptin was significantly decreased in CS induced COPD rat model compared with those in model group (p < 0.05 Figures 9(b) and 9(d)). 40 mg/kg baicalin was also markedly increased ACTH level as compared to control (p < 0.05, Figure 8(a)). There was no change in CRH level after treating with baicalin (p>0.05, Figure 9(c)), indicating that baicalin regulates HPA axis function to some degree. It was also found that there was no significant difference in ACTH, corticosteroid, and leptin between model group and budesonide treated group (p > 0.05).

## 4. Discussion

We have previously reported the adequate COPD rat model with advanced stage clinical features induced by 36-week side stream cigarette smoke exposure [19]. In the present study, we applied the same method to establish COPD rat model and evaluated the effects of baicalin on COPD. Compared to similar studies, our research has several advantages, such as the fact that 36-week side-stream smoke exposure rat model was used for inducing and mimicking stable and advanced stage COPD clinical features and wide histopathological analysis by using small airways, pulmonary small arteries, and lung parenchyma. Furthermore, we used blood serum, BALF, and lung tissue for systematic inflammatory factor inspection. HPA axis function was also determined for better understanding the mechanism of action of baicalin in the treatment of COPD.

We have not bothered to look up the reference that cigarette smoke is one of the main encountered risk factors for COPD and it is associated with accelerated decline in FEV and a higher mortality rate when compared to patients with other diseases [20, 21]. Therefore, COPD model criteria were based on lung function decline and pathological changes of lung tissue [22]. Our results showed that lung function decline and distinct pathological changes were observed in CS induced rat model, indicating that rat model of COPD was successfully established. We have also found that baicalin can ameliorate lung function and improve pathological changes and indicators, such as inflammation scores and destruction index; mean linear alveolar indexes were improved in different level compared with model group, demonstrating the lung function protecting effects of baicalin.

COPD is largely associated with systemic and chronic inflammation of airway and lung parenchyma, which increases in course of acute exacerbations [23]. Thus, inflammatory factors were also measured in blood serum, BALF, and lung tissue; we universally evaluated the effects of baicalin on the regulation of inflammatory factors.

Inflammatory mediators including IL-1*β*, IL-6, IL-8, and TNF-*α* have an important effect on air way inflammation in COPD and amplify the inflammatory response and act as proinflammatory factors [24, 25]. A study's results indicated that cigarette smoke treatment significantly reduces IL-4 and IL-6 and promotes CCL3 and CCL8 expressions in mast cells [26]. It has been also found that the levels of IL-1*β* in the induced sputum and lung of COPD patients were significantly increased when compared with nonsmokers [27]. System cells such as macrophages, mast cell, neutrophils, and B lymphocyte [28] and TNF-*α* can elicit the synthesis of IL-8 and activate T lymphocytes [29]. Levels of IL-17 and other Th17 cytokines are also increased in sputum and airways of patients with COPD and might play a role in orchestrating neutrophilic inflammation in the lungs [30]. Regulatory T cells (Tregs) have been found to play a vital role in the development of COPD. In airways, Tregs function as anti-inflammatory factors secretion and recruitment of other anti-inflammatory cells [31, 32]. During inflammation proinflammatory mediators are antagonized by anti-inflammatory cytokines [33]. Treg related interleukin-10 (IL-10), the anti-inflammatory cytokine inhibitory factor, antagonizes the action of the major inflammatory cytokines [34, 35]. Studies showed that IL-10 level was decreased in COPD patients while healthy volunteers expressed higher level of IL-10 [36]. Therefore, lung function and inflammation can be improved by decreasing inflammatory factors [37]. Tregs were differentiated for ameliorating inflammatory effect through TGF-*β*1 in COPD against the Th17 proinflammatory effect, preserving inflammation balance [32]. Our results showed the same trend in the above mentioned studies, 36 CS exposure increased IL-1*β*, IL-6, IL-8, IL-17, TGF-*β*, and TNF-*α* level in CS induced COPD rat model, and baicalin reduced proinflammatory mediators and increased anti-inflammatory cytokine level, IL-10, both in blood serum, BALF, and lung tissue. These results suggest that baicalin improves lung function and reduces inflammatory conditions by regulating proinflammatory and anti-inflammatory factor imbalance.

Matrix metalloproteinases (MMPs) are regulatory proteases which are the extracellular matrix (ECM) remodellers characterized by their substrate specificity to degrade ECM proteins. Many of MMPs are activated by smoking or oxidative stress [38]. In recent studies more focus has been centered on MMP-9. MMP-9 is not produced by lung resident cells; it is produced by various forms of stimulation. Studies have revealed that levels of MMPs, especially MMP-9, are elevated in both sputum and BALF from patients with COPD and healthy smokers [39]. In the early onset of emphysema/COPD mice had increased influx of inflammatory cells and upregulation of MMPs, such as MMP-2, MMP-9, and MMP-12 in the lung [40]. Our results also showed increased MMP-2 and MMP-9 level in COPD model rat serum, which decreased after baicalin treatment implying baicalin improves airway remodelling in rat model of COPD. TIMPs are the inhibitors of MMPs, preventing MMPs from cleaving ECM components. Study has shown that COPD patients expressed lower level of TIMP-1 [22]. By contrast, our results showed increased TIMP-level in COPD rats which decreased after baicalin treatment, contrary to the above mentioned results, indicating that TIMP-1 content in lung tissue should be evaluated for further analysis. VEGF is also a biomarker of alveolar destruction and contributes to small airway remodelling and angiogenesis [41, 42], and VEGF level increased in the sputum of smokers [43]. And our results are consistent with published reports. Baicalin also reduced increased VEGF level in COPD rat model, indicating that baicalin prevents alveolar destruction and displays anti-airway remodelling effects in the rat model.

Oxidative stress has an important role in the pathogenesis of COPD [44]; oxidative damage induces lipid peroxidation of membrane phospholipids and accelerates MDA production and antioxidant enzymes inactivation [45]. Antioxidant enzymes, SOD, repair cells and reduce damage; SOD and GPx activity is lower in COPD patients compared to nonsmokers [46]. It was also reported that GPx activity is even lower in severe COPD patients than moderate COPD patients, and its activity is directly correlated with FEV [47]. Heme-oxygenase1 (HO-1) is the inducible stress protein that implicates a cytoprotective role against the toxic agents [30]. It is known that HO-1 is a protective mediator in CS-induced COPD [48]. It was reported that HO-1 ameliorates smoke induced lung emphysema by reducing inflammatory mediators and oxidative damage and by decreasing inflammatory cell recruitment [49]

MDA reflects the degree of damage induced by oxidative stress, which is the result of the reactive oxygen species and lipid oxidation [50]. MDA increased in COPD patients and model due to oxidative stress. Our results found that MDA significantly increased after CS exposure in COPD model while SOD and GSH-Px and HO-1 significantly reduced, suggesting that lipid peroxidation accelerated, and free radicals are increased concomitantly. However, baicalin treatment could attenuate these biological parameters and increase total antioxidant capacity of COPD rats, suggesting that baicalin improves CS-induced COPD condition by inhibition oxidative stress.

Studies showed that in severe COPD patients HPA axis function was suppressed; thus cortisol level decreased which reflects lower anti-inflammatory activity and therefore could have led to an increased risk of reexacerbation which led to increased glucocorticoid use [51]. It is believed that corticotropin releasing hormone (CRH) is secreted by the hypothalamus, which accelerates production of adrenocorticotropic hormone (ACTH) from the pituitary. ACTH stimulates the release of cortisol through exciting the adrenal cortex, which in turn has a negative feedback on CRH and ACTH [52, 53]. Our results showed that baicalin can improve HPA axis by increasing ACTH, cortisol, and leptin level.

Blood gas is an important indicator of respiratory function [54]. In severe COPD patients, arterial blood gas analysis demonstrates a life-threatening episode that needs close monitoring or intensive care [55]. Therefore, in severe stage COPD patients, blood gas indices should be analyzed. In the present study, significant change was found in CS induced rats blood gas related indicators compared to controls, and blood gas indices in baicalin treated rats improved significantly.

Body maintains healthy states when it keeps balance between inflammatory and anti-inflammatory mediators; when this balance shifts toward inflammatory mediators (including cells like Th17 and cytokines), organ or tissue or cells will trigger inflammatory responses. Therefore restoring the imbalances between airway-inflammation and anti-airway inflammation is one of the strategies to treat COPD. In the present study, we further proved that baicalin exerts anti-inflammatory and anti-remodelling effect on cigarette smoke induced COPD rat model. Baicalin has lung function protection role in CS induced COPD rat model by regulating proinflammatory and anti-inflammatory factors and proairway remodelling and anti-remodelling factors in blood serum, lung tissue, and BALF. Baicalin also increases antioxidant capacity of CS induced rat model of COPD.

Smoking cessation is considered to be the most cost-effective intervention method to reduce the risk of developing COPD and to reduce its progression; 3 months of smoking-cessation intervention indicated marked improvements in lung-function parameters in subjects who completely quit smoking [56–58]. However, the majority of smokers are unable to quit smoking due to addiction to it, and accelerated lung function decrease and disease progression will occur after long years of cigarette smoking. Here we reported the rat model of COPD simultaneously exposed to cigarette smoking and intervened with baicalin. And baicalin contributes to lung function increase and regulation of imbalances between inflammatory and anti-inflammatory factors. These results demonstrated that baicalin may be a potential novel drug for the treatment of COPD. However some clinical studies should be conducted for revealing its mechanism of action and for providing solid basis for a new drug development.

## Data Availability

All data and materials used in the present study are available from the corresponding author upon reasonable request.

## Supplementary Materials

Supplementary Materials Supplemental Figure 1: effects of baicalin on VEGF and TGF-*β* concentration. (A) VEGF; (B:) TGF-*β*. The data are shown as the mean ± the standard deviation (SD). ^*∗*^p < 0.05 and ^*∗∗*^p < 0.01 indicate a statistically significant difference compared to the model group; ^#^p < 0.05 and ^##^p < 0.01 indicate a statistically significant difference in model group compared with control group. VEGF, vascular endothelial growth factor; TGF-*β*, transformation growth factor.
