# Supplementary material for: Baicalin Exerts Anti-Airway Inflammation and Anti-Remodelling Effects in Severe Stage Rat Model of Chronic Obstructive Pulmonary Disease
Source: Evid Based Complement Alternat Med. 2018 Oct 8;2018:7591348. doi: 10.1155/2018/7591348 (PMC6196890; doi:10.1155/2018/7591348)
Supplement: Supplementary Materials — Supplemental Figure 1: effects of baicalin on VEGF and TGF-β concentration. (A) VEGF; (B:) TGF-β. The data are shown as the mean ± the standard deviation (SD). ∗p < 0.05 and ∗∗p < 0.01 indicate a statistically significant difference compared to the model group; #p < 0.05 and ##p < 0.01 indicate a statistically significant difference in model group compared with control group. VEGF, vascular endothelial growth factor; TGF-β, transformation growth factor. [file 7591348.f1.zip › 7591348.f1/mat.7591348.v2.pdf]

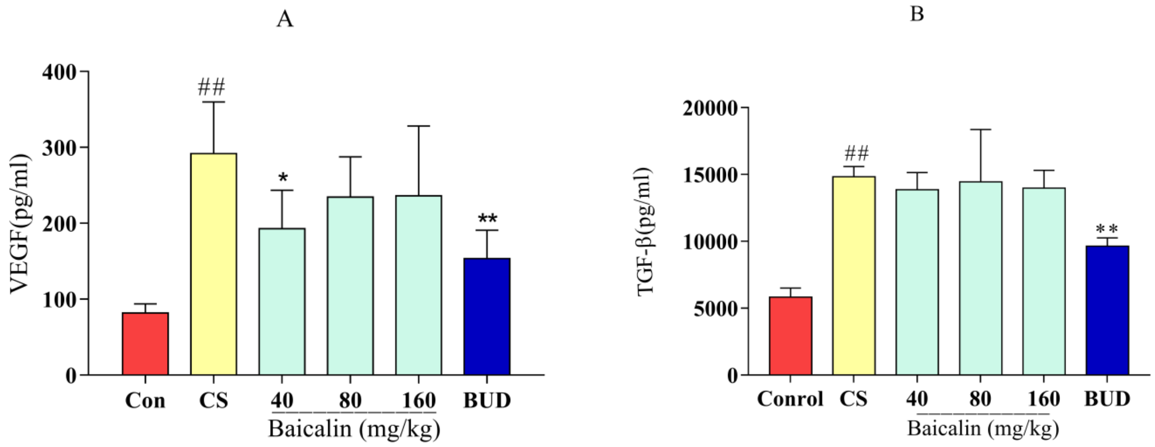

Supplemental Figure 1. Effects of baicalin on VEGF and TGF-β concentration. A:VEGF; B: TGF-β; The data are shown as the mean ± the standard deviation(SD).*$p < 0.05$ and **$p < 0.01$ indicate a statistically significant difference compared to the model group; #$p < 0.05$ and ##$p < 0.01$ indicate a statistically significant difference in model group compared with control group.
